# Meta-dimensional data integration identifies critical pathways for susceptibility, tumorigenesis and progression of endometrial cancer

**DOI:** 10.18632/oncotarget.10509

**Published:** 2016-07-09

**Authors:** Runmin Wei, Immaculata De Vivo, Sijia Huang, Xun Zhu, Harvey Risch, Jason H. Moore, Herbert Yu, Lana X. Garmire

**Affiliations:** ^1^ Molecular Biosciences and Bioengineering Graduate Program, University of Hawaii at Manoa, Honolulu, HI, USA; ^2^ Epidemiology Program, University of Hawaii Cancer Center, Honolulu, HI, USA; ^3^ Harvard School of Public Health, Harvard University, Boston, MA, USA; ^4^ Yale School of Public Health, Yale University, New Haven, CT, USA; ^5^ Institute for Biomedical Informatics, Perelman School of Medicine, University of Pennsylvania, Philadelphia, PA, USA; ^6^ Department of Biostatistics and Epidemiology, Perelman School of Medicine, University of Pennsylvania, Philadelphia, PA, USA

**Keywords:** endometrial cancer (EC), GWAS, data integration, pathways, data mining

## Abstract

Endometrial Cancer (EC) is one of the most common female cancers. Genome-wide association studies (GWAS) have been investigated to identify genetic polymorphisms that are predictive of EC risks. Here we utilized a meta-dimensional integrative approach to seek genetically susceptible pathways that may be associated with tumorigenesis and progression of EC. We analyzed GWAS data obtained from Connecticut Endometrial Cancer Study (CECS) and identified the top 20 EC susceptible pathways. To further verify the significance of top 20 EC susceptible pathways, we conducted pathway-level multi-omics analyses using EC exome-Seq, RNA-Seq and survival data, all based on The Cancer Genome Atlas (TCGA) samples. We measured the overall consistent rankings of these pathways in all four data types. Some well-studied pathways, such as p53 signaling and cell cycle pathways, show consistently high rankings across different analyses. Additionally, other cell signaling pathways (e.g. IGF-1/mTOR, rac-1 and IL-5 pathway), genetic information processing pathway (e.g. homologous recombination) and metabolism pathway (e.g. sphingolipid metabolism) are also highly associated with EC risks, diagnosis and prognosis. In conclusion, the meta-dimensional integration of EC cohorts has suggested some common pathways that may be associated from predisposition, tumorigenesis to progression.

## INTRODUCTION

Endometrial cancer (EC) arises from the endometrium, the inner lining of the uterus. It is the most prevalent gynecologic malignancy and one of the most common cause of deaths of women's cancers. In 2016, it is estimated that there will be 60,050 new cases of EC, and an estimated 10,470 people will die of this disease in the US. Patients with later stage EC have higher risks of tumor recurrence and lower 5-year survival rates (Stage I and Stage II 69% - 88%, Stage III and Stage IV 15% - 58%) [1]. To seek genetic causality of EC, a series of genome-wide association studies (GWAS) have been conducted recently [2–4], with the focus on the association between single nucleotide polymorphisms (SNPs) and EC, however, inconsistencies abound in these studies.

Besides the lack of replicability exemplified by the EC studies above, there exist other issues of SNP-based GWAS approach. For example, highly significant SNPs usually can only explain a small proportion of heritability while moderately significant SNPs may also harbor predictive information [5–7]. Set-level GWAS is an appropriate approach to allow the consideration of effects from the moderately significant SNPs. It summarizes SNPs within certain common biological function sets, such as pathways, and then identifies the disease-associated sets. This design enables us to better uncover the genetic architectures of complex traits, as well as better understanding of the disease mechanisms [8]. Various methods for pathway-level GWAS analysis have been explored [9–12]. Based on the testing hypotheses, they can be categorized into two groups [10]: 1) Competitive hypothesis, which compares variants or genes within a given pathway with those outside of this pathway. 2) Self-contained hypothesis, which only considers elements in pathways of interest and compares them to the null (non-associated) genomic background [13].

Beyond genetic predisposition, other alteration events including somatic mutation and gene expression changes also have consequences on the development of EC [14, 15]. The idea of integrating different data types from multiple domains has been applied before [15–18]. Somatic mutation, the acquired mutations on the germline genetic background, can be measured by technologies such as exome-Seq. Gene expression at the transcriptome level can be measured via RNA-Seq platform. Furthermore, survival analysis enables the assessments of the prognostic value of pathways [19, 20]. Beyond the gene-level, pathway-level statistical tests have been applied to gene expression data type and prognosis analysis [19, 20]. By cross-comparison the results among various data types, we can better evaluate pathways through different stages of EC.

In this study, we first conducted pathway-level GWAS analyses of EC with four different methods, and combined their results by Monte-Carlo simulations [21]. Accordingly, we selected 20 top pathways based on their combined p-values. A further stratification analysis on EC patients based on these pathways identifies two subgroups. To evaluate the overall importance of these 20 pathways across EC development stages, we sequentially performed pathway-level analyses on exome-Seq somatic mutation, RNA-Seq gene expression, and survival analysis from The Cancer Genome Atlas (TCGA). We integrated results across these four data types based on average rankings and confirmed the significance of some cancer-associated pathways, such as p53 signaling and cell cycle pathways. Moreover, we also reveal that other pathways, including IGF-1/mTOR signaling, IL-5, and sphingolipid metabolism may also be overall highly relevant to EC susceptibility, diagnosis and prognosis.

## RESULTS

### GWAS analysis on EC data

We first conducted SNP-level analysis, however, obtained no single SNP with genomic-control corrected p-value (GC-P) < 1e^−8^ (Supplementary Figure 1). Seven SNPs exceeded the moderate threshold of GC-P < 1e^−5^ (Supplementary Figure 1, Supplementary data 1). They are rs6707690, rs11735301, rs919927, rs1830175, rs4725999, rs4594727, and rs4689789. These SNPs lie within the following four genes: CTNNA2, SORCS2, GBX1, and ZNF676. We then tested the interactions among top 50 SNPs, using Multifactor Dimensionality Reduction (MDR) [22, 23]. Among them, rs6707690 (CTNNA2), rs2019978 (CASP3) and rs9541072 (LOC105370246) showed most significant interactions, and was ranked the best in six out of ten times of cross-validation (empirical P = 0.0099). Caspase-3, encoded by gene CASP3, can cleave β-catenin in the process of cellular apoptosis [24]. CTNNA2 encodes α-catenin and was reported as a tumor repressor gene frequently mutated in head and neck cancers [25]. Our result suggests that the SNPs in CTNNA2 and CASP3 may have a synergistic effect on EC risk. Next, we performed the gene-level GWAS analysis and obtained more than 800 significant genes with empirical P < 0.05 (Supplementary data 2). Genes of interest are discussed later within the context of pathways of interest.

**Figure 1:**
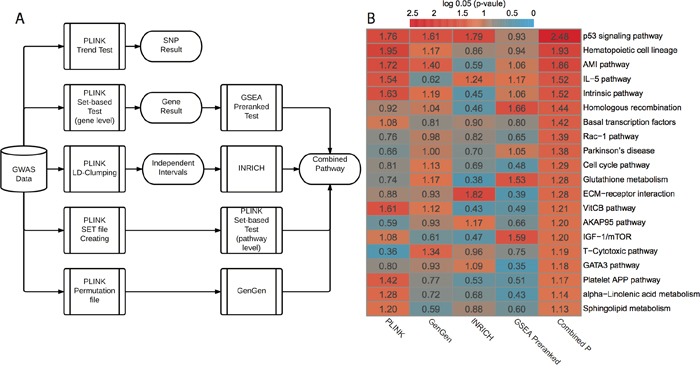
GWAS workflow and pathway-level results **A.** Workflow of GWAS analysis. PLINK trend test is applied for basic association test at SNP level. PLINK set-based test is applied for gene-level and pathway-level analysis. Additionally, three other pathway-level analysis methods are used: GSEA Pre-ranked test, INRICH and GenGen. **B.** Results from four different pathway-level computational methods and combined p-values. All p-values are log_0.05_ transformed and a log_0.05_ transformed p-value greater than 1 is significant with p-value < 0.05.

We subsequently conducted pathway-level GWAS analyses (Figure 1A). Briefly, we applied four pathway-level packages that are based on either competitive hypothesis or self-contained hypothesis: PLINK set-based test [11], INRICH [9], GenGen [10] and GSEA Pre-ranked test [12]. In each method, we employed permutations to generate empirical p-values (or nominal p-values in GSEA). We used a total of 403 KEGG and BIOCARTA pathways for pathway-level annotations. In all, PLINK set-based test, GenGen, INRICH and GSEA Pre-ranked test gave 20, 24, 11 and 20 significant pathways with empirical P < 0.05, respectively (Supplementary data 3). The correlation matrix of p-values based on the four methods is shown in Supplementary Figure 2. Since the best pathway-level association method is still controversial, we combined these p-values using Monte-Carlo simulations (10,000 times). We chose the top 20 pathways (all combined P < 0.05, Figure 1B) for subsequent analyses. The scatter plot based on the average vs. standard deviation (SD) of rankings across four methods shows that the top 20 pathways have high rankings consistently, regardless of the computational methods (Supplementary Figure 3).

To investigate if our results are confounded by other factors, we performed a series of evaluations. First, we compared the pathway size (number of genes) with combined p-values and obtained no strong correlation (Pearson's r = 0.03, Supplementary Figure 4A). Next, in the light that genes heavily connected in the network tend to have more SNPs measured on common genotype platforms [26], we examined the likelihood that genes with higher SNP frequencies are annotated by more pathways. We did not see a clear relationship between the SNP frequencies and the pathway annotation degree among genes at either original or log scales (Pearson's r = 0.08, Supplementary Figure 4B). Thirdly, we investigated the relationship between the average SNP frequency of each gene and gene-level p-value and we obtained no clear relationship at the original or log scales (Pearson's r = 0.02, Supplementary Figure 4C). Finally, to investigate if the top 20 pathways tend to have higher average SNP frequency (0.202) than the expectation, we used a bootstrap resampling method to estimate the background distribution of the average frequency of SNPs among 20 random pathways. The bootstrap p-value is 0.4155 (Supplementary Figure 4D), demonstrating that the top 20 pathways do not have significantly higher SNP frequencies. Therefore, we conclude that the GWAS results are reliable.

We further categorized the top 20 pathways into six functional super-groups (Supplementary data 1), based on BRITE hierarchy of KEGG and BIOCARTA pathway category. These super-groups are: cell signaling and cell cycle regulation, metabolism, immunology, blood coagulation, genetic information processing, and others. As expected, some pathways belong to more than one functional group. Among them, cell signaling and cell cycle regulation includes the largest number (seven) of pathways, and p53 signaling pathway is most significant (combined P = 0.0006, Figure 1B). Alteration of processing genetic information is commonly observed in cancers, especially the deficiency of DNA repairing. This is reflected by homologous recombination, with a significant combined P = 0.0135. The link between the deficiency of immune system and cancer has been well established [27], and here it is evident by the statistical significance in immunology related pathways, such as IL-5 pathway (combined P = 0.0105). We also observed sphingolipid metabolism difference in our result. Sphingolipid metabolism is one of the lipid metabolic pathways that have been related to cancers [28].

**Figure 2:**
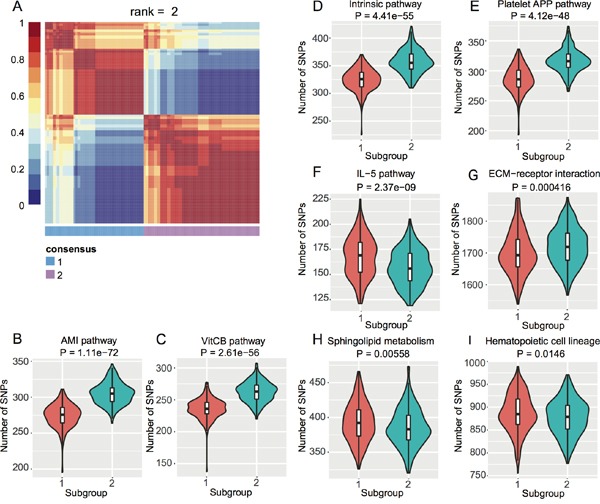
NMF-based subgroups of EC GWAS cohort **A.** Heat map of consensus matrix when rank = 2, where two consensus subgroups are observed. **B.** ~ (I) Violin plots of each pathway with t-test P < 0.05 between the two subgroups from (A).

To investigate if there are subpopulations among the EC GWAS cohort, we conducted Non-negative Matrix Factorization (NMF) based clustering using the normalized SNP counts of the top 20 pathways. We identified two subgroups of EC patients (Figure 2A) based on consensus clustering (Cophenetic Coefficient = 0.91). We then checked which pathways contributed to this stratification by student's t-test. We obtained eight pathways with P < 0.05 (Figure 2B–2I) and interestingly, four blood coagulation pathways gave the highest significance (Figure 2B–2E). Blood coagulation abnormalities were reported in cancer patients and even affect the therapeutic options [29–34].

**Figure 3:**
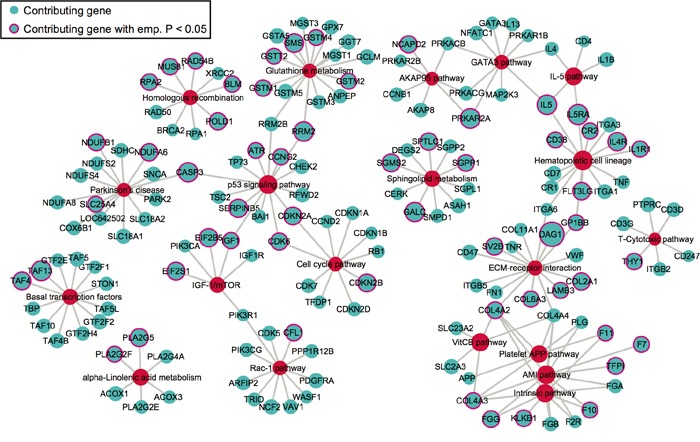
The network of top 20 pathways and their genes Genes (blue) are mapped from contributing SNPs in PLINK set-based test. The significance level of a gene or pathway (red) is represented by the size of the node, based on the log_0.05_ transformed. Genes with pink outlines are significant with empirical p-value < 0.05.

We next extracted the contributing SNPs of top 20 pathways based on PLINK results and mapped them to genes. Based on the pathway- and gene-level results (Supplementary data 2), we created a pathway-gene mapping network using Cytoscape [35]. Such network provides a direct depiction of the connections among top 20 pathways by common genes (Figure 3). As expected, pathways within the same super-group tend to connect with each other, such as the blood coagulation pathways.

### Pathway-level analysis of TCGA exome-Seq data

We were interested to see if any pathway with an increased susceptibility to EC also had preferable somatic mutations [36]. To study this, we investigated EC exome-Seq data from TCGA. We used MutSigCV [37] to identify gene-level significance of somatic mutations, and then performed GSEA Pre-ranked test to conduct pathway-level significance analysis. A total number of 12734 genes were used for GSEA Pre-ranked test, and 415 genes were included in top 20 pathways. Among these pathways determined by the GWAS analysis, three have FDR < 0.05: p53 signaling pathway, rac-1 pathway and IGF-1/mTOR. IL-5 pathway also has FDR < 0.1 (Table 1, Supplementary data 3). All these four pathways belong to the cell signaling and cell cycle regulation super group (Supplementary data 1). Interestingly, IGF-1/mTOR pathway in our study overlaps with PI3K/AKT pathway, one of the most frequently mutated pathways in EC reported by TCGA [15].

**Table 1:** GWAS top 20 pathways and their significance levels in other data sets

Pathway	GWAS Comb. P	exome-Seq FDR	RNA-Seq FDR	Survival FDR
p53 signaling pathway (KEGG)	0.0006	0.036356	0.02015	0.027883
Hematopoietic cell lineage (KEGG)	0.0031	0.652337	0.542707	0.068472
AMI pathway (BIOCARTA)	0.0038	0.837747	0.11669	0.31534
IL-5 pathway (BIOCARTA)	0.0105	0.083992	1	0.024749
Intrinsic pathway (BIOCARTA)	0.0106	0.741155	0.399023	0.151369
Homologous recombination (KEGG)	0.0135	0.600056	0.11669	0.162814
Basal transcription factors (KEGG)	0.0143	0.856576	0.820071	0.806224
Rac-1 pathway (BIOCARTA)	0.0154	0.001747	0.090195	0.556147
Parkinson's disease (KEGG)	0.016	0.297392	0.541898	0.71187
Cell cycle pathway (BIOCARTA)	0.0207	0.389954	0.029553	0.033771
Glutathione metabolism (KEGG)	0.0216	0.974187	0.307288	0.310803
ECM-receptor interaction (KEGG)	0.0217	0.849114	0.762493	0.066176
VitCB pathway (BIOCARTA)	0.0266	0.311884	0.084054	0.406027
AKAP95 pathway (BIOCARTA)	0.0274	0.838687	0.250959	0.05903
IGF-1/mTOR (BIOCARTA)	0.0275	0.000913	0.461597	0.165714
T-Cytotoxic pathway (BIOCARTA)	0.0287	0.82339	1	0.04847
GATA3 pathway (BIOCARTA)	0.0289	0.179809	0.155	0.713263
Platelet APP pathway (BIOCARTA)	0.0301	0.526359	0.0806	0.254728
alpha-Linolenic acid metabolism (KEGG)	0.0325	0.433548	1	0.289216
Sphingolipid metabolism (KEGG)	0.0342	0.391808	0.175326	0.171003

### Pathway-level analysis of TCGA RNA-Seq data

Our next question was whether the pathways subjective to EC-susceptibility also have measurable changes in gene expression, such as those shown by eQTL studies [38]. To answer this, we utilized TCGA RNA-Seq data from paired tumor/adjacent normal tissues. We used paired samples, instead of the population-based tumor and normal control cohort, to reduce the noise in the RNA-Seq data [39]. We applied *Pathifier* algorithm to obtain pathway deregulation score (PDS) for each pathway within each patient, as reported before [19]. We then performed pairwise permutations (tumor/adjacent normal) on PDS, by randomly assigning the paired PDSs to pathways. This allowed us to obtain empirical p-values of pathways, followed by FDR based multiple-hypothesis testing. Among the GWAS top 20 pathways, two of them have FDR < 0.05: p53 signaling pathway and cell cycle pathway. Additionally, three pathways have FDR < 0.1: rac-1 pathway, VitCB pathway and Platelet APP pathway (Table 1, Supplementary data 3). Both VitCB and Platelet APP pathways are in blood coagulation super-group.

### Pathway-level analysis of TCGA relapse-free survival data

**Figure 4:**
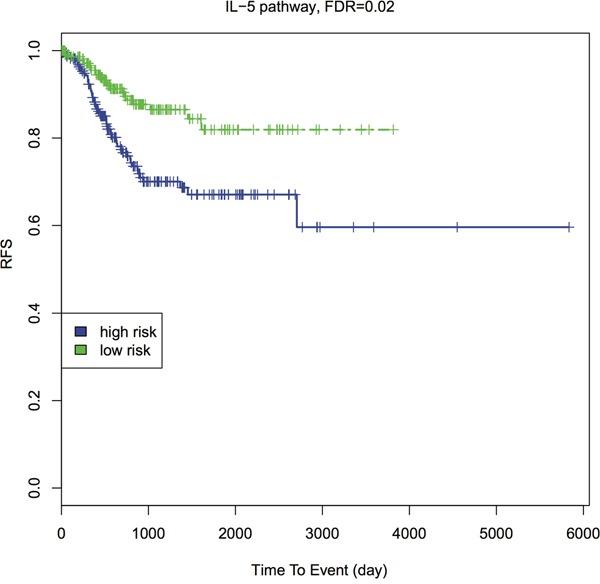
Kaplan-Meier survival curves of IL-5 pathway with FDR Patients are dichotomized by the median PDS into higher- vs. lower- risk groups. The Wilcoxon log-rank p-value is calculated to detect the survival difference between these two groups, then adjusted by FDR.

A more downstream potential impact of genetic predisposition pathways is cancer patient's relapse-free survival (RFS) [40]. To explore this, we conducted pathway-based RFS analysis using all EC primary tumor RNA-Seq data. Similarly, we transformed the gene-based RNA-Seq data to a pathway-based PDS matrix and then performed individual RFS analysis. We dichotomized the patients into high-risk (higher PDS) and low-risk (lower PDS) groups, by the median PDS. We used Kaplan-Meier curves to present RFS of the high- and low-risk groups (Figure 4 and Supplementary Figure 5). The survival difference between the two groups is calculated by Wilcoxon log-rank p-value, followed by FDR-based multiple hypothesis testing. Interestingly, among top 20 GWAS pathways, four separate the patients into higher vs. lower risk groups with FDR < 0.05: p53 signaling pathway, cell cycle pathway, IL-5 pathway and T-Cytotoxic pathway. Three additional pathways have FDR < 0.1 (Table 1, Supplementary data 3). IL-5 pathway gives the most significant result (FDR = 0.0247, Figure 4). The importance of IL-5 is justifiable since previous studies showed that IL-5 enhanced cancer invasion and migration [41, 42].

### Integrative analysis of all four data types

**Figure 5:**
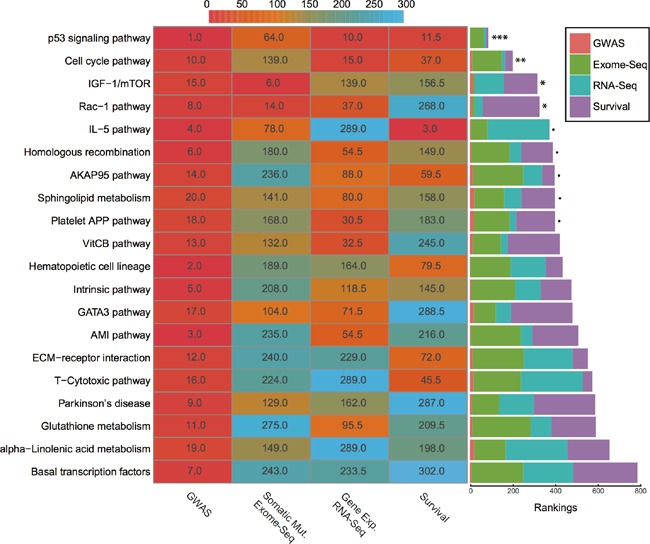
Rankings of top 20 GWAS pathways across different data sets (Left) Heat map refers to pathway rankings in four different data sets. (Right) The corresponding stacked bar plot of each pathway. *** P < 0.001; ** P < 0.01; * P < 0.05; P < 0.1 from the permutation test.

**Figure 6:**
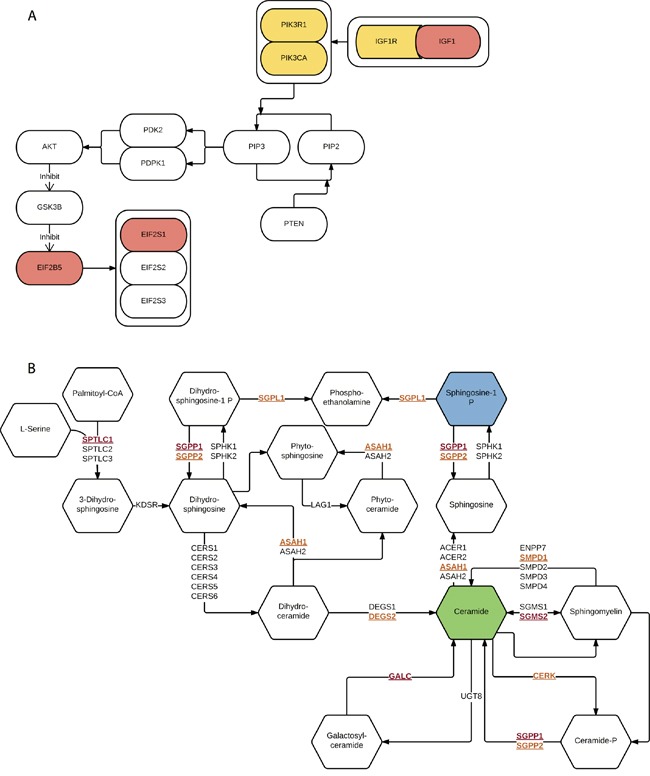
Illustration of two representative pathways **A.** IGF-1/mTOR. Genes mapped by contributing SNPs (yellow), genes mapped by contributing SNPs and also with empirical P < 0.05 (red). **B.** Sphingolipid metabolism. Genes mapped by contributing SNPs (yellow and underline), genes mapped by contributing SNPs and also with empirical P < 0.05 (red and underline), survival factor metabolite (blue) and apoptosis factor metabolite (green).

Before integration, we first ranked all pathways based on the results from four different data types mentioned above. Then we calculated the average rankings followed by the permutation test. The permutation-based empirical p-value represents the overall consistency of pathway significance across different EC data types (Supplementary data 4). Anchoring on the top 20 pathways obtained from GWAS analysis, we observed four pathways with empirical P < 0.05 and five pathways < 0.1 (Figure 5 and Supplementary data 4). Impressively, p53 signaling pathway achieves the most consistent highly rankings across all data types, followed by cell cycle pathway and IGF-1/mTOR (Figure 5). IGF-1/mTOR plays critical roles in the regulation of cell proliferation, survival and energy metabolism [43]. Six genes (mapped by contributing SNPs) contributed in this pathway majorly and three of them are significant in the gene-level analysis: IGF1, EIF2B5 and EIF2S1 (Figure 3, Supplementary data 2). Figure 6A shows the topological relationship of these genes: IGF1 can activate AKT and further influence the eukaryotic translation initiation factor 2′ isoforms (encoded by genes including EIF2B5 and EIF2S1). Sphingolipid metabolism shows an overall consistency empirical p-value of 0.0986 across the four data types, and the topological pathway view is illustrated in Figure 6B. Two metabolites in this pathway, sphingosine-1 phosphate (S1P) and ceramide, are well studied in cancer development [44]. S1P acts as a survival factor whereas ceramide as a tumor-suppressing factor. Three genes (SGPP1, SGMS2 and GALC) that encode the metabolic enzymes of these two metabolites are significant at the gene level (Figure 3, Figure 6B).

## DISCUSSION

### Meta-dimensional data integration to study genotype-phenotype relationship

Our study here represents an exemplification of using systems-genomics approach to interrogate molecular mechanisms of diseases [18, 45]. Comparing to other studies, this study is unique in several aspects. First, we investigated data generated from different populations and different data types: GWAS from one cohort, and somatic mutation exome-Seq, tumor-adjacent tissue RNA-Seq, relapse-free survival from TCGA. Secondly, we have implemented a pathway-based modeling scheme for each data type, so that biological significance of pathways is straightforwardly demonstrated. Thirdly, we developed a model-based integration framework, where each data type is individually modeled and then integrated at the post-model statistical level. This framework is different from other methods such as CONEXIC [46] and PARADIGM [20], which are restricted to data generated from the same cohort. Rather, our model-based integration can be applied to various data sources [18].

We hypothesize that some pathway alterations that predispose the population to EC may be manifested at the somatic levels and impact the disease progression [47, 48], since it was observed that 40% of genes predisposing the population to cancers are frequently mutated at the somatic level [49]. Under this assumption, we initiated our study by first investigating the EC risk susceptibility and focused on pathway-level analysis. We avoided the bias in each pathway-level GWAS analysis method by combining the results from four different computational methods using Monte-Carlo simulations. This approach generates more conservative yet less biased results than simple procedures such as Fisher's combined tests. We also conducted pathway-level analyses on other omic data types: somatic mutation, tumor/adjacent gene-expression. Although the cases and controls are different given different cohorts (a very frequent dilemma that researchers face), our previous studies provide evidence that information aggregated on the pathway level can overcome the barriers of different omics data types, and perhaps is a generic approach to integrate different omics data sets [19, 50].

### Relevance of detected top pathways to the etiology of endometrial cancer

We utilized average rankings with permutation-based empirical p-values to measure the overall significance of the top 20 pathways across different data types. Cell signaling and cell cycle regulation related pathways are ranked top four (Figure 5, Supplementary data 4). One pathway that stands out as significant in all data types is p53 signaling pathway. This is not surprising, as TP53 accounts for one of the highest mutation rates in most cancers, including EC [15, 51]. And CASP3 in this pathway was found with high significance, corresponding to our MDR analysis result. Interestingly, caspase 3 was reported to mediate the stimulation of tumor cell repopulation during cancer radiotherapy [52]. The second most significant pathway, cell cycle pathway, is significant in all other analyses, except exome-Seq data. This is also expected, as cell cycle dysregulation is a hallmark of cancer [53, 54].

IGF-1/mTOR is significant in both GWAS and exome-Seq data. In this pathway, IGF1 and its downstream targets, EIF2S1 and EIF2B5 are significantly associated with EC (Figure 6A). IGF1 plays an important role in the growth of multiple tumors as well as in the prevention for cells from apoptosis [55]. Supporting our findings, other studies also reported that the level of IGF1 was associated with endometrial cancer as well as other cancer types [56–58]. In addition, we also observed genes mapped by contributing SNPs, such as PIK3CA, PIK3R1, and IGF1R, which were reported as commonly mutated genes in EC on somatic level by TCGA [15]. Drugs that target IGF-1/mTOR pathway, such as mono-clone antibodies for IGF-1R, may have therapeutic values in EC treatment [59–62].

Other pathways that may be of therapeutic targets are IL-5 pathway and sphingolipid metabolism. IL-5 pathway gives significant results in GWAS, survival data, and has FDR < 0.1 in exome-Seq data. The survival result shows its important value in EC relapse prognosis. The binding of IL-5 to IL-5Rα receptor induces the migration of cancer cells was reported [41], and corresponding genes showed significant results in our GWAS analysis. On the other hand, few studies discussed sphingolipid metabolism alterations in EC specifically [63], although the relationship between sphingolipid metabolism and other cancers was well-studied [64]. Here we have identified that this pathway and multiple genes that are involved in ceramide and S1P metabolism (e.g., GALC, SGPP1, and SGMS2) also potentially predispose the individuals to EC risks. Sphingolipid metabolism is not only likely to contribute to EC progression and chemoresistance [63], but also associated with higher risks at the genetic level.

Perhaps most surprising finding of this study is that four blood coagulation pathways clumped together in GWAS network can significantly differentiate the two subgroups among EC patients. Blood coagulation disorders were observed in some of the cancer patients before [31–34], and it is worth paying attention to especially considering its influence on therapeutic treatments [29, 30]. A recent study has shown direct evidence that patients with myoma and especially those with endometrial cancer have hypercoagulability [65]. We thus suspect that genetics may explain part of the observed hypercoagulability. Several genes, such as F7 and F10 were significant in GWAS results (Figure 3), and may lead to susceptibility to EC. The binding of F7 to tissue factor (F3) initiates the blood coagulation cascade [66] and F3 plays a critical role in cancer development [32, 34]. On the other hand, elevating of F10 protein was observed in clinical cancer cases as well as cell lines [67].

### Conclusions and future work

Our meta-dimensional, pathway-level analysis offers a systematic way of studying the influence of genetic variants in EC, through multiple sources of information that include GWAS, exome-Seq, RNA-Seq, survival analysis. It has confirmed the importance of most well-studied cancer-associated pathways, such as p53 signaling pathway and cell cycle pathway. Moreover, it also sheds light on some less well-studied pathways in relation to EC, such as IGF-1/mTOR, sphingolipid metabolism, and IL-5 pathway. The impacts of these pathways may be associated with EC susceptibility, tumor development, and progression. It would be interesting to conduct follow-up population studies to investigate the association between these pathways and EC risks. Additionally, pathway level stratification in EC patients identified blood coagulation as the top functional super-group differentiating the subpopulations. It will be of great interest to confirm this in other cohorts, as well as associating it with other clinical outcomes. From the study design point of view, a comprehensive study in the future which obtains different types of data from the same population will likely yield more consistent pathway-level results. Some limitations of the pathway-based integration include the incomplete knowledge about pathways currently, as well as lacking topology information in the current pathway metrics. One alternative method is to derive functional units using the graph-based network approach [20]. Nevertheless, we speculate that by marrying prior biological knowledge with the powerful integration of meta-dimensional data sets, it is possible to better understand the etiology of diseases such as EC.

## MATERIALS AND METHODS

### Data sets

#### GWAS data

We obtained data from Connecticut Endometrial Cancer Study (CECS) [68], a population-based case-control study on 668 incident cases with type I endometrial cancer and 674 population controls. DNA samples were genotyped using the HumanOmniExpress BeadChips (Illumina, Inc., San Diego, CA). We filtered the SNPs with completion rates < 90%, minor allele frequencies < 1%, and Hardy–Weinberg equilibrium violation of p-value < 0.0001 in controls. Subsequently, 482 cases and 571 controls with 649,351 SNPs were selected for the GWAS analysis [69]. The race composition is dominantly white, accounting for 1044 out of 1053 samples.

#### Exome-Seq data

We downloaded TCGA public Illumina GA_DNASeq level 2 data sets from Broad Institute (BI). A total number of 194 patients with both primary solid tumor samples and normal samples (4 tumor adjacent normal samples and 190 blood samples) were used in the analysis. Only the variants unique to the tumor but not the normal sample of each patient were used for downstream analysis.

#### RNA-Seq data

We downloaded TCGA public IlluminaGA_RNASeqV2, and IlluminaHiSeq_RNASeqV2 data sets from University of North Carolina (UNC). We used 23 pairs of tumor/adjacent normal samples for pathway-level gene expression analysis. Additionally, we used 457 primary tumor samples with their clinical information for pathway-level survival analysis (downloaded on 03/24/2015).

#### Pathway data

We downloaded pathways from Molecular Signature Database (MsigDB) (http://www.broadinstitute.org/gsea/msigdb/index.jsp) [12]. In this study, we considered 186 KEGG and 217 BIOCARTA pathways in total. To further characterize the functional super-groups of pathways, we used KEGG BRITE hierarchy (http://www.genome.jp/kegg/brite.html) and BIOCARTA pathway category (http://www.biocarta.com/genes/index.asp).

### GWAS analysis

#### SNP-level association analysis

We used Cochran-Armitage trend test for the association analysis between the single locus and the disease, by taking different genotypes into consideration. To test the SNP-SNP interactions associated with EC, we used Multifactor Dimension Reduction (MDR) [22, 23], a 10-fold cross-validation will be conducted to get a cross validation consistency of SNP pairs and permutation test to calculate their significant levels.

#### Gene-level analysis

We used PLINK set-based test for the GWAS data at the gene level. First, we created set files, which contain SNPs associated with a particular gene based on their locations (within +/−20 kb of the gene body). To minimize the bias from correlated SNPs in the same set, we employed linkage disequilibrium (LD) prune so that SNPs under consideration are relatively independent. We selected top 5 (default setting) SNPs with p-values ≤ 0.05 to represent the genes. We used the average chi-square statistics of selected SNPs to represent the set statistics. To obtain the empirical p-values for sets, we applied permutations (N = 10,000) on the phenotype labels. After each permutation, pseudo statistics were re-calculated for the SNPs as well as the SNP set statistics. The empirical p-values were determined as the percentages of permuted set statistics exceeding the original statistics.

#### Pathway-level analysis

Four pathway-level packages were used for this purpose: PLINK set-based test, GenGen, INRICH and GSEA Pre-ranked test. We elaborate the details of each method below:

Pathway-level PLINK set-based test summarizes N SNPs per set (pathway). Given that a smaller N leads to a larger number of significant pathways, we examined the effect of a series of Ns on the pathway outcomes, and determined the optimum N = 20, as it is the elbow point (the point of changing slopes) based on pathways vs. N (Supplementary Figure 6). Like gene-level analysis, we associate SNPs to genes within 20 kb upstream/downstream of gene bodies (window length). We performed 10,000 permutations on the case/control labels.

GenGen is a software which extends Gene Set Enrichment Analysis (GSEA) [12] on the GWAS data. It maps the most significant SNP to each gene and applies the GSEA approach to summarize the pathway-level result. We considered all pathways within the size interval of [1, 500] and performed 1,000 permutations on phenotype labels. All other parameters were set to default values.

INRICH takes a set of independent genomic intervals created by PLINK. It calculates the primary enrichment statistic for each pathway, depending on the number of intervals that overlap at least one target gene [9]. We used PLINK LD-based clumping (threshold 0.5) to create the interval data. We set the significance thresholds of index SNPs (p1) as 0.01 and clumped SNPs (p2) as 0.05. We ran 10,000 times of permutations by randomly assigning intervals to genomic locations, with the constraints that each null interval r_i_ ⊠ R approximately matches the original interval I_i_ ⊠ I (i = 1, …, k) for the number of SNPs and overlapping genes; we also ensured approximately similar SNP densities per kb.

GSEA Pre-ranked test was adapted from the gene-level results of PLINK set-based test. We removed the genes contain no significant SNP (empirical P = 1), then sorted genes in the descending order of log_0.05_ transformed empirical p-value. We conducted GSEA Pre-ranked test based on gene ID permutations.

As solid evidence of methodological superiority is lacking [13], we calculated combined p-values based on a Monte-Carlo simulation method (http://comisef.wikidot.com/tutorial:correlateduniformvariates), which also allowed us to control the false positive rates. We first applied Pearson's correlation test on the results from these four methods to generate the correlation matrix A, and then performed Choleski decomposition (A = L^′^L) on the correlation matrix A. We then ranked all pathways by their p-values across methods and calculated their average rankings. Using rankings instead of p-values ensures all methods being treated equally [21]. We simulated (N = 10,000) the null p-values from a uniform distribution [0, 1], while keeping the correlations of four methods by multiplying the upper-triangle matrix L. Next we calculated the combined p-value per pathway as the percentages of simulated average rankings that surpassed the original average rankings.

#### Evaluation of potential confounding factors

To check if our GWAS results were confounded by other factors, such as pathway size and gene size, we performed a series of evaluations on different factors. Pathway size is the number of genes in a pathway and gene size is the number of SNPs in a gene. SNP frequency is calculated as SNP counts per 1kb of gene length. Pathway annotation degree of a gene is the number of pathways this gene is assigned to. We also estimated the significance level of the average SNP frequency in top 20 pathways, by comparing to the simulated null-distribution of average SNP frequency in random 20 pathways (bootstrap 10,000 times).

#### Pathway-based subpopulation identification

We used the R-package NMF [70] to identify subpopulations in the GWAS EC patient data. For each patient, we summed all SNPs to pathway level in the top 20 pathways, and applied Z-score normalization for each pathway. Since NMF requires non-negative values, we subtracted the minimum value for the entire matrix. We determined the optimal number of subgroups by Cophenetic Coefficient. We then performed student's t-test between the subgroups to identify significant pathways.

#### Data availability

The raw genotype data are submitted to the Epidemiology of Endometrial Cancer Consortium (E2C2) under NCI (National Cancer Institute), which has initiated the data submission to dbGaP (accession number assigned phs000893.v1). Additionally, the pre-computed p-values of SNPs are on the website: garmiregroup.org/data. Other processed data can be found in Supplementary data.

#### Network visualization

We extracted contributing SNPs of top 20 pathways from PLINK results and mapped them to genes. Corresponding empirical p-values were also extracted from PLINK results. To visualize the relationship of genes and pathways, we used Cytoscape [35] to build a customized network, where the log_0.05_ (empirical P) are represented by the size of the nodes.

### TCGA exome-Seq somatic mutation analysis

We applied MutSigCV [37] to the EC somatic mutation data from TCGA. We used full coverage file and calculated the background mutation rate per gene. We then conducted a log_0.05_ transformation of the p-values of genes generated by MutSigCV, and removed the genes with log_0.05_ p-value equal to 0 (original p-value = 1), since they didn't contribute meaningful mutations. We then sorted the genes and used them for GSEA Pre-ranked test at the pathway level result.

### TCGA RNA-Seq gene expression analysis

For pathway-level analysis on the paired TCGA RNA-Seq data, we first applied DESeq2 [71] to normalize the expression among samples. We then used *Pathifier* [72] algorithm to transform gene-level expression information into pathway-level information, represented by pathway deregulation score (PDS) per patient. Personalized PDS is derived from the concept of “principal curve” [73]. It is a measurement of degrees of deviation from the “normal status” along the principal curve. We calculated the paired t-statistics on the original PDS matrix for each pathway. Then, we conducted pairwise permutation tests on the PDS matrix by randomly reassigning PDS for each patient, where the pair information was kept intact during permutation. The null hypothesis for this pairwise-permutation test (coupling the tumor/adjacent tissue from the same patient with the same pathway assignment) is that the PDS obtained from a particular pathway is the same as PDS generated from other pathways which are reassigned randomly. We ran 10,000 permutations to obtain the empirical p-values for each pathway, and adjusted all p-values by FDR.

### Survival analysis based on the TCGA RNA-Seq data set

Relapse-free survival analysis was used to study the prognostic associations of pathways based on EC TCGA RNA-Seq data set. A total of 537 samples had clinical information, and among them 457 samples had relapse free survival information (downloaded 03/24/2015). Patients without relapse events during the study were considered censored. To realize pathway-level survival analysis, we first applied *Pathifier* to summarize the expression values from the gene level to pathway level. We then dichotomized the patients by median PDS, and assigned the patient with a higher PDS than median to a higher-risk group; otherwise, we assigned this patient to a lower-risk group. We used Kaplan-Meier curves to present the prognosis of the higher- and lower-risk groups. The Wilcoxon log-rank test was then conducted on the Kaplan-Meier curves to detect the survival difference between these two groups. All survival analysis was conducted using the R package Survival [74, 75]. All log-rank p-values were then adjusted by FDR.

### Integrative analysis of top pathways

To evaluate the overall significances of the top 20 pathways, we ranked them based on their results from four different data types and calculated the average rankings. We then conducted resampling for 10,000 times on the rankings from each data type, and calculated the average rankings. This generated a null-distribution of average rankings which can be used to calculate the empirical p-value of each pathway. Pathway empirical p-values represent the overall consistency across different data types.

## SUPPLEMENTARY FIGURES AND TABLES










