# Supplementary material for: Meta-dimensional data integration identifies critical pathways for susceptibility, tumorigenesis and progression of endometrial cancer
Source: Oncotarget. 2016 Jul 9;7(34):55249–63. doi: 10.18632/oncotarget.10509 (PMC5342415; doi:10.18632/oncotarget.10509)
Supplement: Supplementary file 1 [file oncotarget-07-55249-s001.pdf]

Meta-dimensional data integration identifies critical pathways for susceptibility, tumorigenesis and progression of endometrial cancer

SUPPLEMENTARY FIGURES

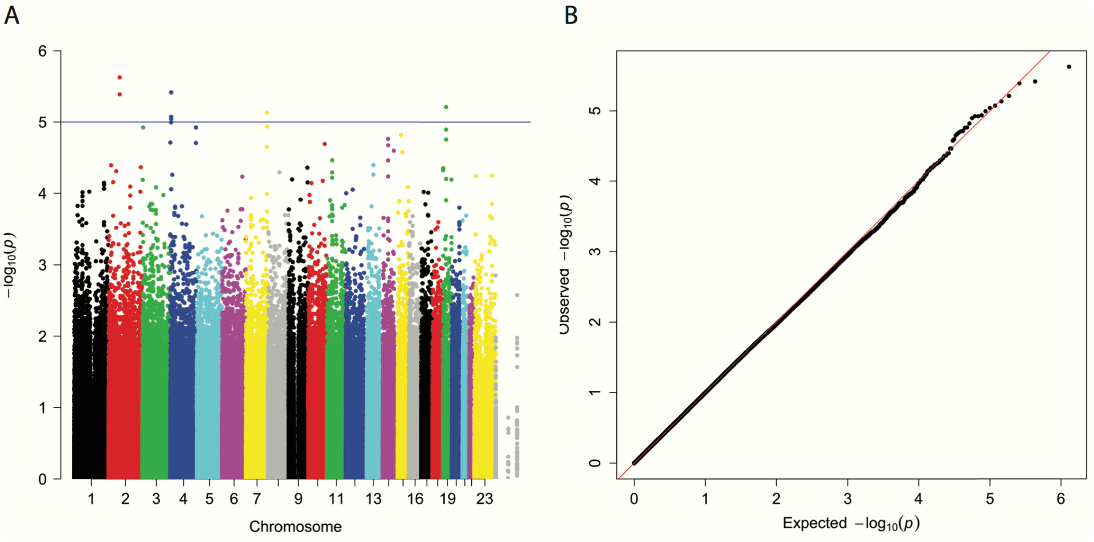

Supplementary Figure S1: SNP-level GWAS results. A. Manhattan plot, genomic coordinates are displayed along the X-axis, with the -$\log_{10}$ (P) for each SNP displayed on the Y-axis **B.** QQ plot, expected distribution of -$\log_{10}$ (P) (X-axis) compared to the observed -$\log_{10}$ (P) (Y-axis).

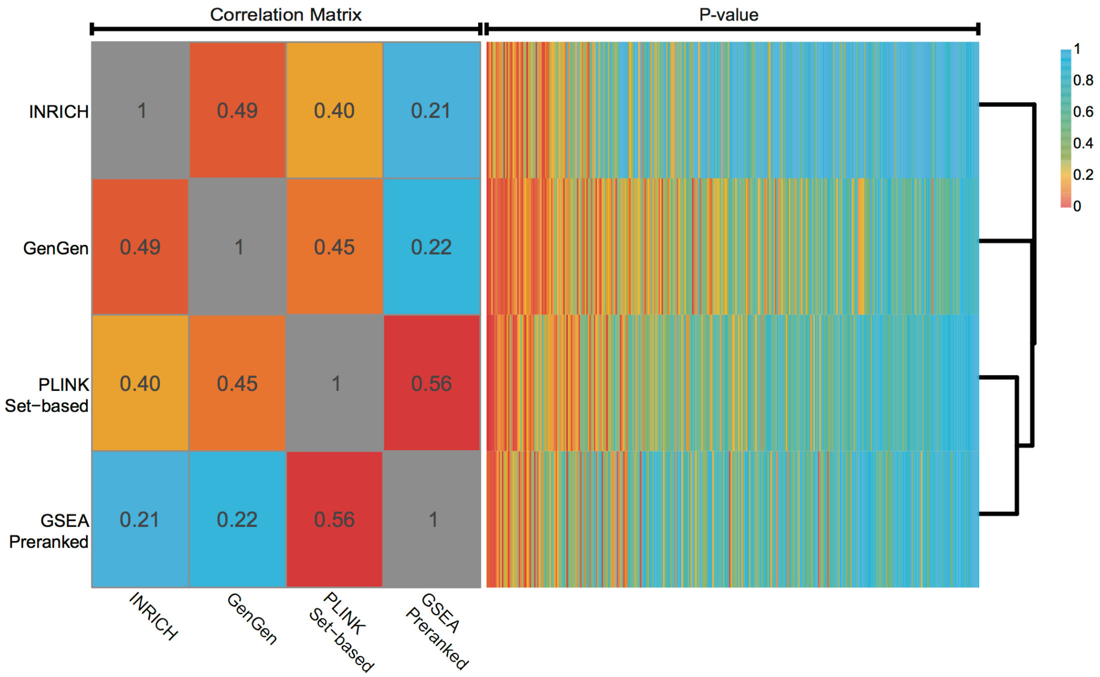

Supplementary Figure S2: Correlation of pathway-level GWAS results across four methods. (Left) Heat map refers to the correlation matrix from different methods. (Right) Heat map refers to the result (p-value) of each method.

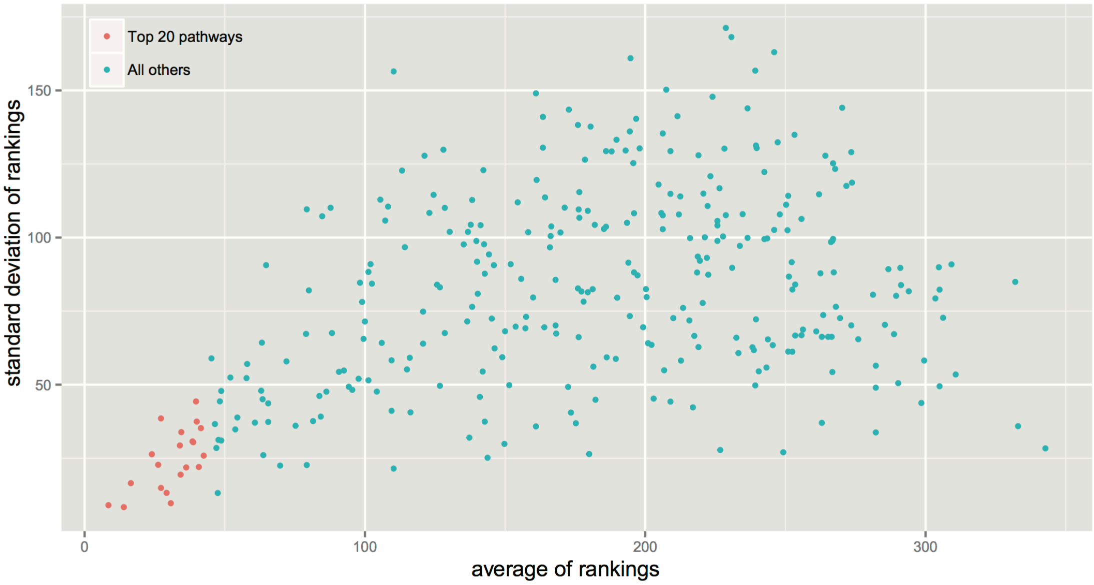

Supplementary Figure S3: The Mean-SD plot of rankings from GWAS pathway analyses. The scatter plot of pathway average rankings vs. standard deviation of rankings. The red points refer to the top 20 pathways we selected by Monte-Carlo simulations.

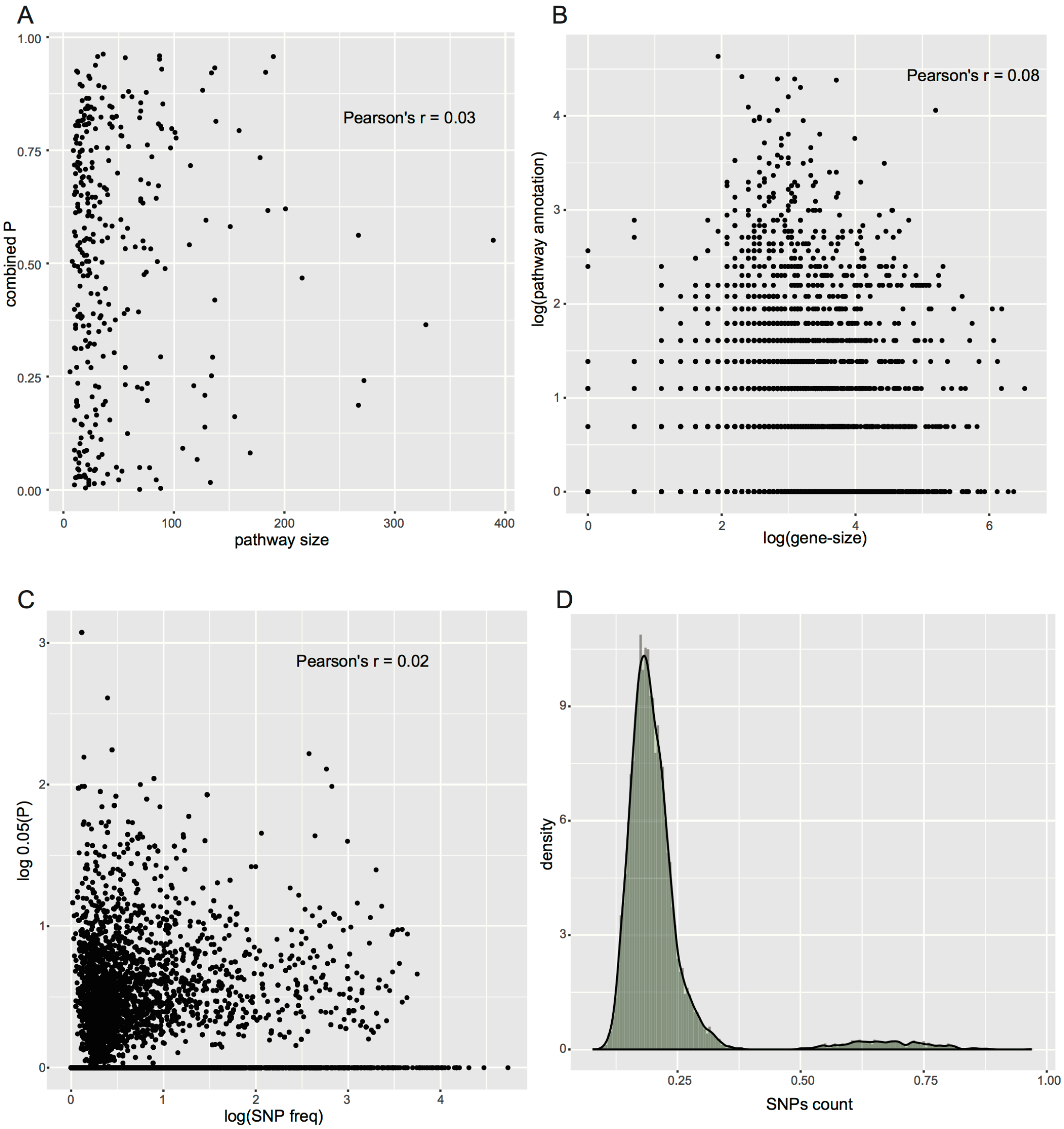

Supplementary Figure S4: Results of the evaluation of potential confounding factors. A. Pathway size vs. Pathway combined p-values **B.** Gene-size vs. Gene-Pathway annotation degree (log-scale) **C.** Gene-SNP frequencies vs. Gene-level p-values (log-scale) **D.** A bootstrap distribution of average SNP frequency in random 20 pathways.

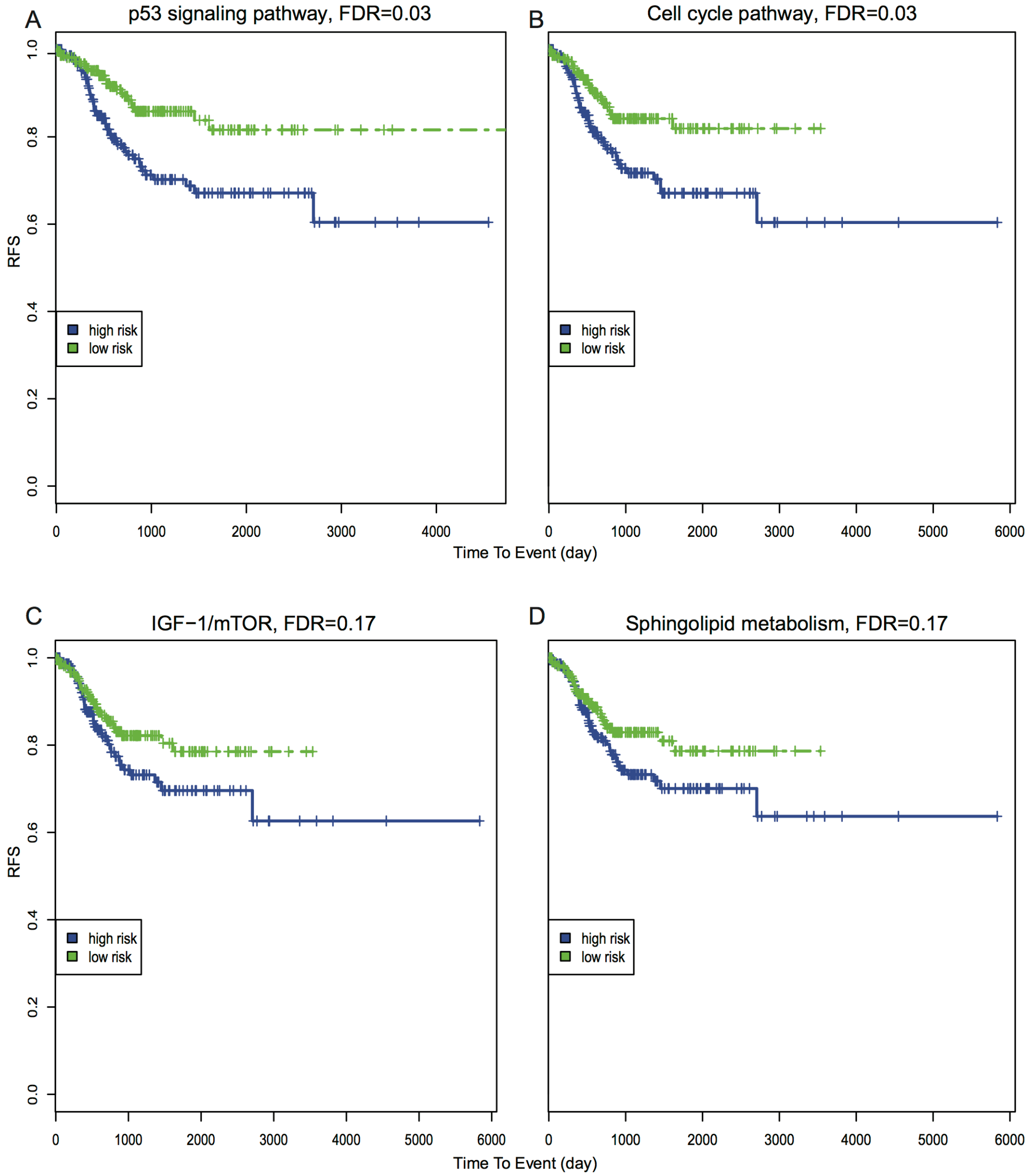

Supplementary Figure S5: Kaplan-Meier survival curves with FDR. A. p53 signaling pathway **B.** Cell cycle pathway **C.** IGF-1/mTOR **D.** Sphingolipid metabolism.

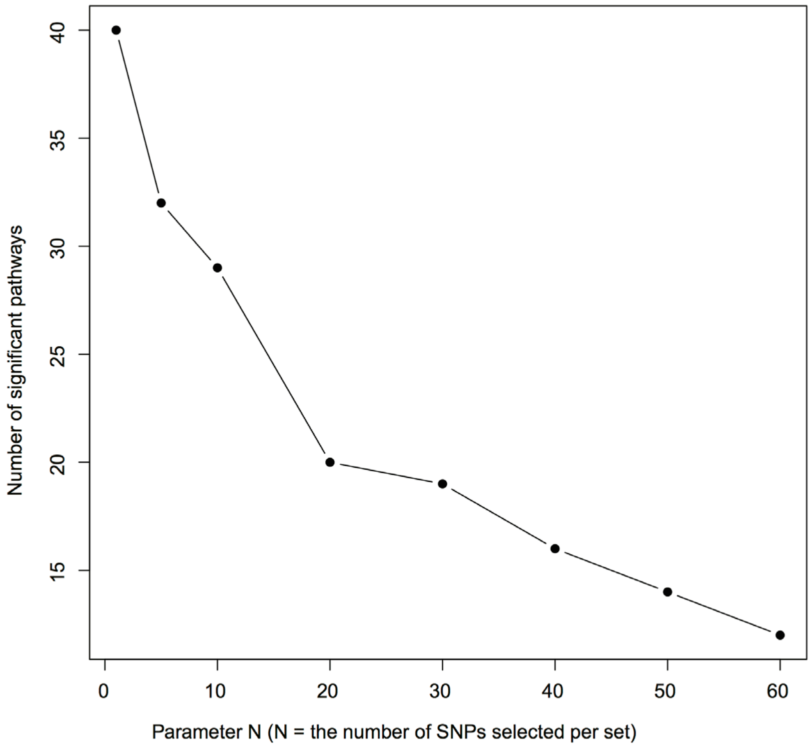

Supplementary Figure S6: Parameter N in PLINK pathway-level analysis vs. the number of significant pathways in results.

Supplementary Data 1:

See Supplentary File 1

Supplementary Data 2:

See Supplentary File 2

Supplementary Data 3:

See Supplentary File 3

Supplementary Data 4:

See Supplentary File 4
